# Resilient and responsive healthcare services and systems: challenges and opportunities in a changing world

**DOI:** 10.1186/s12913-021-07087-8

**Published:** 2021-10-04

**Authors:** Siri Wiig, Jane K. O’Hara

**Affiliations:** 1 SHARE-Centre for Resilience in healthcare, Faculty of Health Sciences, University of Stavanger, N-4036 Stavanger, Norway; 2 School of Healthcare, University of Leeds, Leeds, UK

**Keywords:** Resilient healthcare, Health systems resilience, Adaptive capacity, Learning, Patient safety, Quality

## Abstract

**Background:**

Resilient and responsive healthcare systems is on the agenda as ever before. COVID-19, specialization of services, resource demands, and technology development are all examples of aspects leading to adaptations among stakeholders at different system levels whilst also attempting to maintain high service quality and safety. This commentary sets the scene for a journal collection on *Resilient and responsive health systems in a changing world*. The commentary aims to outline main challenges and opportunities in resilient healthcare theory and practice globally, as a backdrop for contributions to the collection.

**Main text:**

Some of the main challenges in this field relate to a myriad of definitions and approaches to resilience in healthcare, and a lack of studies having multilevel perspectives. Also, the role of patients, families, and the public in resilient and responsive healthcare systems is under researched. By flipping the coin, this illustrates opportunities for research and practice and raise key issues that future resilience research should pay attention to. The potential of combining theoretical lenses from different resilience traditions, involvement of multiple stakeholders in co-creating research and practice improvement, and modelling and visualizing resilient performance are all opportunities to learn more about how healthcare succeeds under stress and normal operations.

**Conclusion:**

A wide understanding of resilience and responsiveness is needed to support planning and preparation for future disasters and for handling the routine small-scale adaptation. This collection welcomes systematic reviews, quantitative, qualitative, and mixed-methods research on the topic of resilience and responsiveness in all areas of the health system.

## Background

Resilience and the capacity to adapt and respond to challenges and changes at different system levels, is fundamental for healthcare services and systems to maintain critical functions and deliver high quality care services across varying conditions [1–3]. Since the beginning of the COVID-19 pandemic, we have all witnessed health systems and service providers worldwide under extreme strain. Healthcare practitioners, managers, policy makers, patients and the public have all had to suddenly, and dramatically, adapt to this new threat to public health, whilst also attempting to maintain their safety and the safety of services. This very visible, tangible expression of the concept of resilience has led to an equally visible increased interest in the concept of healthcare system resilience. Indeed, it is evident that whilst many people might not know of, or understand the concept, almost everyone’s lives have been touched by, and contributed to, the resilience of our global public health effort.

A cursory look at the social media feeds of people working in healthcare services will tell you that there has been a range of impacts of the pandemic on services, and not all of them negative. Healthcare practitioners have described huge adaptations to service delivery, and the impacts that these have had professionally and personally. What is evidently of utmost importance now for the academic as well as healthcare community, is to explore, document and understand these adaptations. What worked and why? What supported these adaptations to happen quickly and safely? What were barriers or challenges for adaptation? What adaptations have, or should have, been retained? What were the knock on effects of the adaptations for services, patients and staff? How have patients, families and the public contributed to the resilience of healthcare services? What does this mean for resilient healthcare theory?

These are just some of the questions that we as a collective community of practitioners and scholars need to address. However, these questions also sit within the wider context of the existing discourse on, and empirical evidence for resilient healthcare theory and practice. The purpose of this collection is to provide a platform for exploration of these questions and debates, to build new knowledge, refine theory, and nurture innovative approaches that might be sustained into, or inform safety management within, a post-pandemic healthcare landscape. In this opening commentary we outline what we regard to be the main challenges and opportunities facing those seeking to document, explore, operationalize or develop resilient healthcare theory and practice globally.

## Main text

### Challenges

There is a myriad of definitions of resilience coming from diverse research fields and sectors [2, 4, 5]. However, a common aspect relates to the capacity to identify and handle disruptions, large or small, and invoke mechanisms for the systems to ‘bounce back’ and establish or reestablish a ‘new normal’ situation [1, 4, 6]. Resilient performance is achieved through a combination of absorption of these challenges, and adaptation and transformation to continue operations when facing disruptions [3]. Disruptions come in varying forms and scales [7]. The disruptions may be positive, such as innovations and favorable new technology that changes and improves work operations [8]. They may also be of more negative character with the potential to cause harm – such as a global pandemic – with significant short- and long-term consequences. However, as yet we have little empirical evidence about how diverse stakeholders both identify, and then respond to this range of disruptions, and how adaptions ensure continued service provision. In particular, insight into the mechanisms behind successful adaptations is of particular interest and importance [9, 10]. This also highlights the role of innovation and learning processes within and across professionals, organizational, and cultural interfaces. We know innovation and collaborative learning are key for resilience, but there is still limited evidence detailing how and why such processes succeed, or not [1, 8, 11].

The rapidly changing world and the societal challenges of infectious disease, economic pressures, pandemics, and continuous rates of adverse events of around 10% of all hospital admissions in the Western world (and even higher in low-income countries), is encouraging the international healthcare system and organizations to look for new approaches [12, 13]. Put simply, there are a significant number of pulls (from inside the healthcare community) as well as pushes (both policy, and the realities and challenges of service delivery) to ‘do safety differently’. Adopting a resilience orientation to these challenges provides us with a new lens through which to view these longstanding, and sometimes seemingly intractable problems.

It is important to state here however, that there is no such thing as “a resilience approach”. Rather, there are diverse resilience approaches which stem from different perspectives, disciplines (e.g. psychology, engineering, ecology) and sectors (health, social science, economics), which collectively may provide new insight into the societal challenges we are facing today. In this collection we encourage a broad empirical orientation on resilience from the smallest team units in service provision [14] to the health systems and actions taken at policy level and on the international scene [2]. We argue that a broad perspective on resilience and responsive healthcare systems, facilitates deeper insights into how systems and actors operate and depend on each other to maintain high quality care. This view is still lacking in the literature and more studies are welcome to identify factors, mechanisms, relations at different system levels [5, 15].

In the resilient healthcare literature [16, 17] the main interest is on complex adaptive systems and a multilevel conceptualization of resilience which depends on stakeholders at different system levels (policy makers, regulators, managers, healthcare professionals, patients). Despite arguing for a systems perspective, studies within resilient healthcare have hitherto mainly focused on how healthcare is provided at the ‘sharp end’ and how frontline healthcare professionals adapt, ‘work around’, or enable things to go well, all the while being surrounded by ever increasing complexity [5, 15, 18]. There is a real need for larger and multilevel studies that investigate how actors at the upper levels of healthcare systems contribute to resilience and create environmental and contextual conditions under which service providers work and perform in resilient ways [19–24].

This also links to the literature on health systems resilience [2]. This literature sees resilience as a broad concept with a multi-sector and multi-level scope. Also, it involves multiple populations in its operationalization. Health systems resilience as a perspective is already heavily drawn upon for solving international health crises. Indeed, it is used by World Health Organization, with some initiatives seeking to translate it into operational indicators aimed at building resilience [2, 6]. Conceptually, the development and use of indicators of resilience as a foundation for assessing and building resilience [25] might be regarded by theoretical purists as being a rather narrow, or fragmented approach. However, this is an area with growing interest and more studies are needed to identify possible empirically driven resilience themes or topics suitable for further development into resilience indicators to guide assessment, performance, and initiatives for establishing interventions and in efforts to build resilience into healthcare services and systems. As described by Barasa et al. [3], there is a paucity of evidence on how to generate or strengthen resilience, as up until recently the literature has been highly conceptual. This marks a call for investigations of what makes systems resilient in the real world, in order to enable critical reflection on strategies and practices for strengthening resilience [3].

One final area we would like to highlight is how resilient performance is co-created as a collective, dynamic responsibility. Indeed, the role of groups, teams, managers, and healthcare professionals in resilience appears to be an important focus for future studies, to tease out more about contextual, structural, relational details of how adaptive capacity is unfolding in healthcare practice and in collaborations across groups and service levels [24, 26, 27]. The same goes for the role of citizens, patients and next of kin in resilience. We know that these groups can take on major responsibility in healthcare – both under normal conditions and during crises – but how we can understand these actors as co-creators and resources in resilient performance is still under investigated [28–31].

### Opportunities

The different challenges we outline above are not meant to be an exhaustive list. Further, if we flip the coin, they constitute opportunities for research and practice and raise key issues that future resilience research should grapple with. The multiple stakeholders involved in creating resilient performance across system levels, and new types of risk and changes (e.g. digitalization of healthcare, security issues, pandemics) open up possibilities for modelling resilience in new ways. Modelling in a complex world is hard, but important for both researchers and practitioners. Modelling increases understanding of the phenomenon, as well as the benefits and limitations of resilience as a scientific or practical approach [32]. Modelling and visualization are important mechanisms for illuminating how systems might operate, and to communicate the sometimes complex message of resilience research to diverse stakeholder groups, as these are both target audiences and key actors for resilient healthcare system and services.

There is also an untapped potential in combining theoretical lenses in resilience research. For example, theoretical approaches in innovation, safety science, psychology, economics, law, political science, and organizational learning can be combined. This will guide studies and interpretation of results in new ways. We also argue there is a potential added value in linking diverse resilience traditions or schools of thought to strengthen learning across these. Resilience Engineering [33] has for example always been multidisciplinary – involving cognitive psychologists, engineers, sociologists – but by combining this school with, for example psychological resilience, could add to the current body of knowledge. Such an approach may advance the understanding of resilience as an emergent phenomenon. Increasing knowledge by drawing on resilience from multiple traditions has the potential to reconcile gaps between individual, team, organizational and system level resilience. We still don’t know how they are linked, and how we can understand these in a holistic way. This is perhaps not a fruitful pathway, but we need research to investigate and potentially reject these possible connections. Finally, there is a call for generating and testing interventions, collaborative tools, and reflexive spaces designed to promote resilience and establish conditions under which resilient performance may occur [1, 18, 34]. Strategies and interventions to strengthen resilience in health systems and in service provision should be research-based. We hope this special collection will contribute to this knowledge generation.

## Conclusion

We encourage a wide understanding of resilience and responsiveness in order to support planning and preparation for future disasters, for adapting to diverse types of system stress, shocks, chronic disturbance, and for handling the routine small-scale adaptation to everyday change [3]. This collection recognizes the aforementioned challenges and opportunities and welcomes systematic reviews, quantitative, qualitative, and mixed-methods research on the topic of resilience and responsiveness in all areas of the health system.

## Data Availability

Not applicable.
